# Dual inhibition of AKT/FLT3-ITD by A674563 overcomes FLT3 ligand-induced drug resistance in FLT3-ITD positive AML

**DOI:** 10.18632/oncotarget.8675

**Published:** 2016-04-11

**Authors:** Aoli Wang, Hong Wu, Cheng Chen, Chen Hu, Ziping Qi, Wenchao Wang, Kailin Yu, Xiaochuan Liu, Fengming Zou, Zheng Zhao, Jiaxin Wu, Juan Liu, Feiyang Liu, Li Wang, Richard M. Stone, Ilene A. Galinksy, James D. Griffin, Shanchun Zhang, Ellen L. Weisberg, Jing Liu, Qingsong Liu

**Affiliations:** ^1^ High Magnetic Field Laboratory, Chinese Academy of Sciences, Hefei 230031, Anhui, P. R. China; ^2^ University of Science and Technology of China, Hefei 230036, Anhui, P. R. China; ^3^ CHMFL-HCMTC Target Therapy Joint Laboratory, Hefei 230031, Anhui, P. R. China; ^4^ Department of Medical Oncology, Dana-Farber Cancer Institute, Harvard Medical School, Boston, MA 02115, USA; ^5^ Hefei Cosource Medicine Technology Co. Ltd., Hefei 230031, Anhui, P. R. China; ^6^ Hefei Science Center, Chinese Academy of Sciences, Hefei 230031, Anhui, P. R. China

**Keywords:** acute myeloid leukemia, FLT3-ITD, AKT, A6745763, FLT3-ligand

## Abstract

The FLT3-ITD mutation is one of the most prevalent oncogenic mutations in AML. Several FLT3 kinase inhibitors have shown impressive activity in clinical evaluation, however clinical responses are usually transient and clinical effects are rapidly lost due to drug resistance. One of the resistance mechanisms in the AML refractory patients involves FLT3-ligand induced reactivation of AKT and/or ERK signaling via FLT3 wt kinase. Via a screen of numerous AKT kinase inhibitors, we identified the well-established orally available AKT inhibitor, A674563, as a dual suppressor of AKT and FLT3-ITD. A674563 suppressed FLT3-ITD positive AML both *in vitro* and *in vivo*. More importantly, compared to other FLT3 inhibitors, A674563 is able to overcome FLT3 ligand-induced drug resistance through simultaneous inhibition of FLT3-ITD- and AKT-mediated signaling. Our findings suggest that A674563 might be a potential drug candidate for overcoming FLT3 ligand-mediated drug resistance in FLT3-ITD positive AML.

## INTRODUCTION

Acute Myeloid Leukemia (AML) is the most common acute leukemia in adults and usually leads to death within months if untreated [1]. The type III receptor tyrosine kinase, FLT3, has been found to play an important role in the differentiation and survival of hematopoietic stem cells in bone marrow [2]. The FLT3-ITD mutation, which is found in approximately 30% of AML patients, has been validated as one of the most important driver oncogenes for AML [3]. A variety of small molecule inhibitors, including the first generation multi-targeted kinase inhibitors, sunitinib, midostaurin (PKC-412), and lestaurtinib (CEP-701), and more selective second generation kinase inhibitors, such as quizartinib (AC220), crenolanib and PLX3397, have been tested in the clinic and have led to initial transient responses but followed by development of resistance [4]. One of the resistance mechanisms in the AML refractory patients is drug treatment-induced elevation of FLT3 ligand levels that can lead to the persistent activation of downstream mediators, such as AKT [5, 6]. We were interested in finding a single agent capable of dually inhibiting AKT and FLT3-ITD kinases; this dual activity characteristic of one small molecule inhibitor would be expected to reduce off-target effects as well as associated potential drug-drug interactions through the standard drug combination approach. Here, we identified the previously reported AKT inhibitor, A674563, as a selective FLT3-ITD inhibitor [7]. A674563 exhibits potent activity against FLT3-ITD positive AML cancer cells *in vitro* and *in vivo* through selective, dual inhibition of AKT and FLT3 kinases, and is also capable of overcoming FLT3 ligand-induced drug resistance in FLT3 wt/ITD co-expressing cells.

## RESULTS

### A674563 exhibits relatively selective anti-proliferative potency against FLT3-ITD-positive AML cancer cells

**Figure 1:**
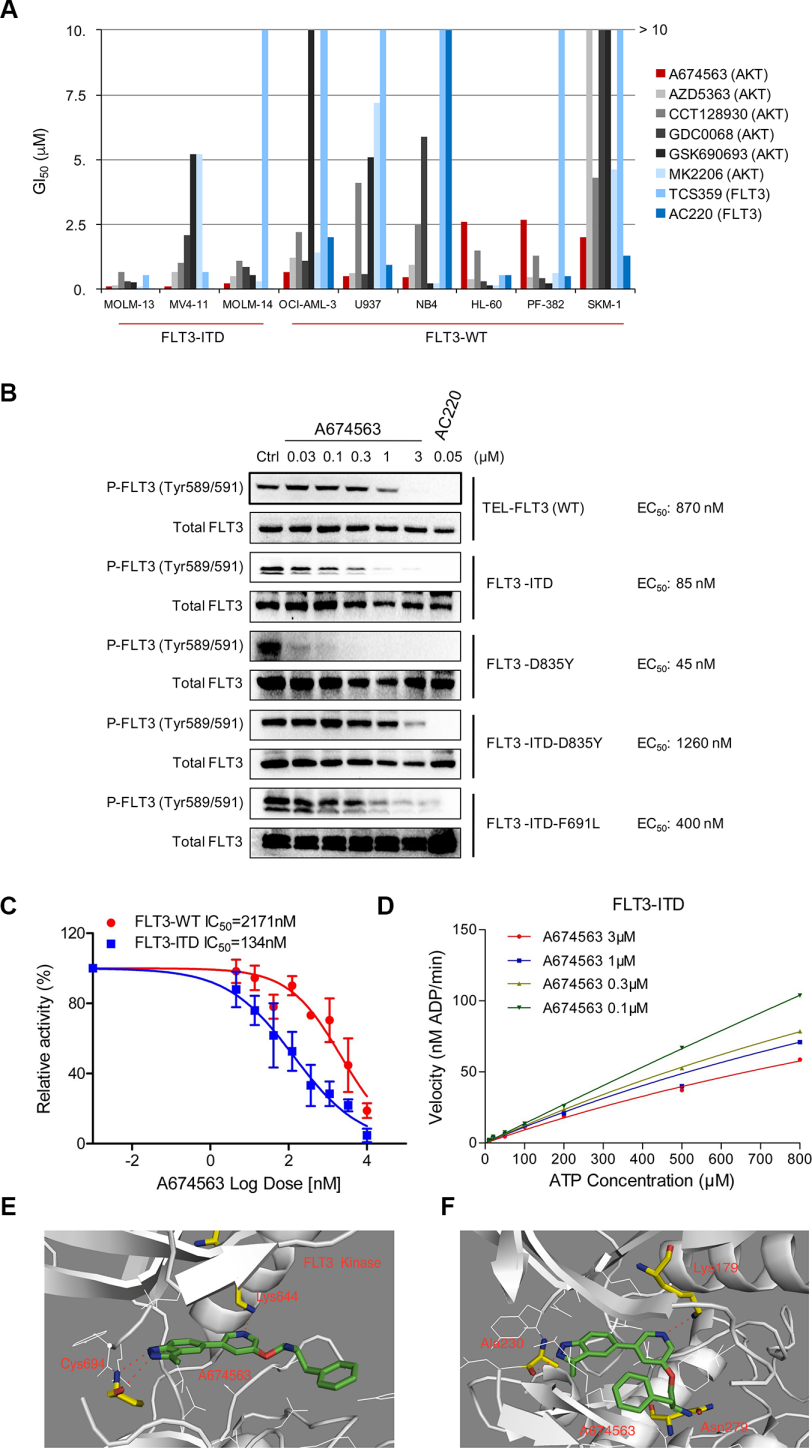
A674563 selectively inhibits FLT3-ITD (**A**) Anti-proliferation effects of AKT inhibitors (A674563, AZD5363, CCT128930, GDC0068, GSK690693, MK2206) and FLT3 inhibitors (TCS359, AC220) against FLT3-ITD positive AML cell lines (MOLM13, MOLM14, MV4-11) and FLT3 wt cell lines (U937, NB4, HL-60, PF-382 and SKM-1). (**B**) Inhibitory Effects of A674563 against auto-phosphorylation of FLT3 wt/mt kinases in the FLT3 wt/mt transformed BaF3 isogenic cell lines. (**C**) Biochemical IC50 determination of A674563 in ADP-Glo assay with purified FLT3-wt (kinase domain) and FLT3-ITD (ITD+kinase domain) proteins. (**D**) Kinetics study with purified FLT3 wt/ITD protein against a range of ATP concentrations. (**E, F**) Molecular modeling illustration of A674563 binding mode in AKT (homology model built upon PDB ID: 1RJB,) and FLT3 (PDB ID: 3CQU) kinases.

We screened a panel of AKT inhibitors using FLT3 inhibitors AC220 [8] and TCS359 [9] as positive controls against FLT3-ITD-positive (MOLM13, MOLM14 and MV4-11) and FLT3 wt (U937, HL-60, PF382, SKM-1, NB4 and OCI-AML3) AML cell lines. (Figure 1A and Table 1) A previously reported AKT inhibitor, A674563, exhibited relatively selective potency against FLT3-ITD-positive cell lines, MOLM13 (GI_50_: 0.06 μM), MOLM14 (GI_50_: 0.18 μM) and MV4-11 (GI_50_: 0.075 μM), versus the FLT3 wt-expressing cell lines (about 5-20 fold less potent). The well-characterized FLT3 kinase inhibitors, AC220 and TCS359, exhibited a similar trend. The clonogenic assay also confirmed the selective efficacy of A674563 against FLT3-ITD positive AML cell lines (MV4-11, EC_50_: 0.092 μM; MOLM13, EC_50_: 0.17 μM; MOLM14, EC_50_: 0.061 μM) compared to FLT3-wt expressing cell lines (PF382, EC_50_: 0.861 μM; U937, EC50: 0.505 μM; HL-60, EC_50_: 0.387 μM) (Supplementary Figure 1).

**Table 1:** A674563 anti-proliferative efficacy against FLT3-ITD positive/wt intact cancer cell lines A

Drug/Cell line GI_50_(μM)	A674563	AZD5363	CCT128930	GDC0068	GSK690693	MK2206	TCS359	AC220
(Primary target)	(AKT)	(AKT)	(AKT)	(AKT)	(AKT)	(AKT)	(FLT3)	(FLT3)
MOLM-13 (FLT3-ITD/wt)	0.06	0.16	0.65	0.29	0.28	0.1	0.56	< 0.0003
MV4-11 (FLT3-ITD)	0.075	0.67	1	2.1	5.2	5.2	0.68	< 0.0003
MOLM-14 (FLT3-ITD/wt)	0.18	0.5	1.1	0.85	0.54	0.32	10-3	< 0.0003
OCI-AML-3 (FLT3-wt)	0.67	1.2	2.2	1.1	> 10	1.4	> 10	3–1
U937 (FLT3-wt)	0.52	0.61	4.1	0.59	5.1	7.2	> 10	0.94
NB4 (FLT3-wt)	0.48	0.93	2.5	5.9	0.22	0.21	> 10	> 10
HL-60 (FLT3-wt)	2.6	0.37	1.5	0.31	0.16	0.16	0.54	0.55
PF-382 (FLT3-wt)	2.7	0.48	1.3	0.44	0.24	0.61	> 10	0.49
SKM-1 (FLT3-wt)	2	> 10	4.3	> 10	> 10	4.6	> 10	1.3

### A674563 displays anti-proliferative activity against FLT3-mutant engineered isogenic BaF3 cells

In a panel of FLT3 kinase wt/mutant–expressing BaF3 isogenic cell lines, A674563 showed potent anti-proliferative activity against the FLT3-ITD mutant (GI_50_: 0.088 μM) compared to parental BaF3 cells (GI_50_: 1.6 μM). (Table 2) Interestingly, it also potently inhibited FLT3-D835Y mutant-expressing cells (GI_50_: 0.045 μM). It was less potent against drug-acquired secondary mutants such as FLT3-ITD-D835Y (GI_50_: 0.42 μM) and FLT3-ITD-F691L (GI_50_: 0.43 μM). In addition, all of the inhibitory activities could be substantially rescued after addition of IL-3, which suggests that inhibitory effects are on-target. (Supplementary Figure 2) However, for the TEL-fused wt FLT3-BaF3 cell line, A674563 exhibited much less potent inhibitory activity (GI_50_: 1.0 μM). We also found that A674563 was most potent against auto-phosphorylation of FLT3-ITD (EC_50_: 0.085 μM) and FLT3-D835Y (EC_50_: 0.045 μM), but was least potent against auto-phosphorylation of FLT3-ITD-D835Y (EC_50_: 1.26 μM), FLT3-ITD-F691L (EC_50_: 0.4 μM) and FLT3 wt (EC_50_: 0.87 μM); this is consistent with the demonstrated anti-proliferative activity of A674563 (Figure 1B and Supplementary Figure 3).

**Table 2:** A674563 anti-proliferative effects against FLT3 wt/mutant transformed BaF3 isogenic cell lines

Cell lines	A674563 (GI_50_: μM)
BaF3-TEL-FLT3-wt	1
BaF3-FLT3-ITD	0.088
BaF3-FLT3-D835Y	0.075
BaF3-FLT3-ITD-D835Y	0.42
BaF3-FLT3-ITD-F691L	0.43
BaF3	1.6

### A674563 selectively inhibits FLT3-ITD mutant kinase biochemically

In the purified enzymatic ADP-Glo assays, A674563 exhibits greater selectivity for the FLT3-ITD mutant (IC_50_: 134 nM) versus FLT3 wt (IC50: 2171 nM). (Figure 1C) Kinetics analysis demonstrates that A674563 is an ATP competitive inhibitor. (Figure 1D) A molecular modeling study of A674563 in FLT3 kinase (PDB ID: 1RJB, homology building) suggests that the NH of indazole moiety forms a hydrogen bond with Cys694 in the hinge binding area, and a nitrogen atom of pyridine moiety in the middle part of the inhibitor forms a hydrogen bond with Lys644. The phenyl ring of the tail part directs into the solvent area and forms a partial π- π interaction with Tyr842. (Figure 1E) For AKT kinase (PDB ID: 3CQU), A674563 forms a similar hydrogen bond in the hinge area with Ala 230 and with Lys179, however the amine group in the tail part forms a third hydrogen bond with Asn 279 and forces the phenyl ring to rotate back to the more hydrophobic ATP binding pocket (Figure 1F).

### A674563 simultaneously inhibits FLT3-ITD- and AKT-mediated signaling

**Figure 2:**
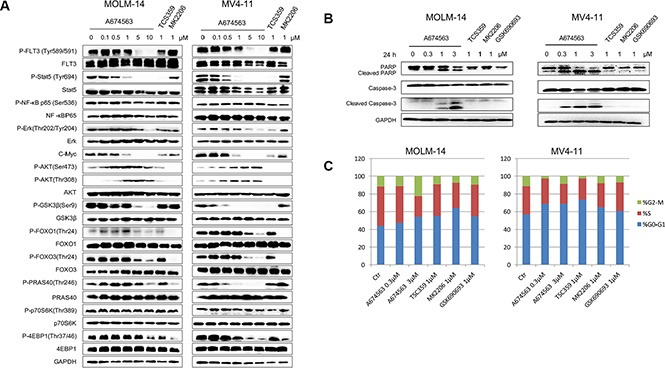
A674563 effect on signaling pathway, cell cycle and apoptosis (**A**) Effect of A674563 on FLT3-ITD mediated signaling in MOLM14 and MV4-11 cell lines. (**B**) Effect of A674563 on apoptosis induction in MOLM14 and MV4-11. (**C**) Effect of A674563 on cell cycle progression in MOLM14 and MV4-11.

We then investigated the effect of A674563 on FLT3- and AKT-mediated signaling pathways in FLT3-ITD-positive cell lines (Figure 2A). A674563 significantly affected the auto-phosphorylation of FLT3-ITD at the Y589/591 site in these intact cancer cell lines starting from 5 μM but potently inhibited the phosphorylation of STAT5, a downstream mediator of FLT3 signaling, as well as the expression of c-MYC starting from 0.5 μM concentration. In contrast, the AKT inhibitor, MK2206, did not affect this signaling pathway [10]. A674563 treatment led to an increase in AKT phosphorylation at both S473 and T308 but displayed inhibitory activity against phosphorylation of signaling molecules downstream of AKT, including GSK3β, FOXO1, PRAS40, and further downstream targets P70S6K and 4EBP1via mTOR kinase, which is consistent with the previous report [11] This suggests that dual FLT3 and AKT inhibition contribute to the growth inhibition of these cells. However, in the FLT3 wild-type (wt) expressing cell lines, OCI-AML-3 and U937, phosphorylation of FLT3 Y589/591 were not detected and phosphorylation of STAT5 as well as cMyc expression were unaffected, except the AKT kinase mediated signaling pathway (Supplementary Figure 4).

### A674563 selectively induces apoptosis and arrests cell cycle progression in FLT3-ITD positive AML cell lines

A674563 induced apoptosis in MV4-11 cells at 0.3 μM and more significantly at 1 μM (Figure 2B). In MOLM14 cells, induction of apoptosis by A674563 was evident at 1 μM, and was more pronounced at 3 μM (Figure 2B). However, even at 1 μM, the selective FLT3 inhibitor, TCS359, and AKT inhibitors MK2206 did not induce apoptosis. In addition, in FLT3 wt-expressing AML cell lines, OCI-AML3 and U937, none of the inhibitors demonstrated a strong apoptotic effect at concentrations up to 1 μM (Supplementary Figure 5). A674563 arrested cell cycle in G0/G1 phase in the FLT3-ITD positive cells, and surprisingly blocked FLT3 wt-expressing cells (OCI-AML-3 and U937) in S phase which suggests a distinct inhibition mechanism (Figure 2C and Supplementary Figure 6).

### Combination of selective AKT and FLT3 kinase inhibitors can mimic the dual inhibitory effect of A674563

**Figure 3:**
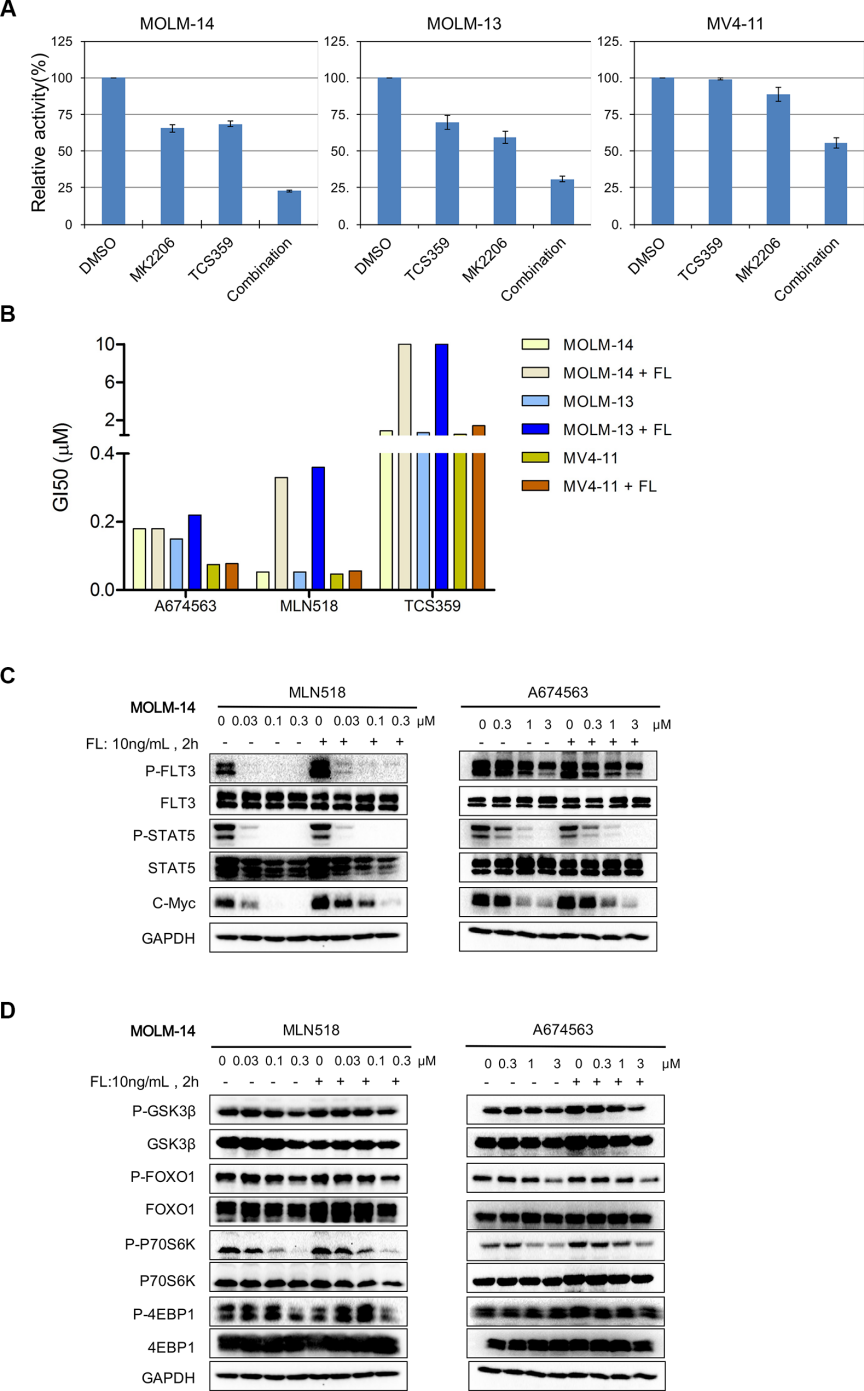
Effect of A674563 on FLT3- and AKT-mediated signaling and overriding of FLT3 ligand-induced drug resistance (**A**) Combination study with FLT3 inhibitor TCS359, and AKT inhibitor MK2206. (**B**) Anti-proliferation effects of A674563 and FLT3 inhibitors, TCS359 and MLN518, against FLT3-ITD-positive AML cells in the presence of 10 ng/mL FLT3 ligand. (**C**) Effect of A674563 and MLN518 on FLT3-mediated signaling in MOLM14 (FLT3-ITD/wt) in the presence/absence of FLT3 ligand. (**D**) Effect of A674563 and MLN518 on AKT-mediated signaling in MOLM14 (FLT3-ITD/wt) in the presence/absence of FLT3 ligand.

We then looked at the combination of AKT and FLT3 inhibitors to see if they were able to recapitulate the effect of dual inhibition by A674563. Due to the high potency of AC220 (single digit nM range) against these FLT3-ITD cells, which would leave little margin to observe synergy with an AKT inhibitor, we chose the less potent FLT3 inhibitor, TCS359, to observe the combination effect more clearly [3]. In the MV4-11 cell line, 1/5 of the GI_50_ concentration (0.12 μM/0.68 μM) of TCS359 combined with 1/50 of the GI_50_ concentration of MK2206 (0.12 μM/5.2 μM) was able to potently and synergistically inhibit the cell proliferation (55% combination inhibition versus 98.96% and 88.64% individual inhibition respectively, CI index: 0.29) (Figure 3A right lane and Supplementary Table 1). Similar synergistic effects were observed with MOLM13 (CI index: 0.41 with 0.12 μM TCS359 and 0.36 μM MK2206) and MOLM14 cells (CI index: 0.49 with 3.3 μM TCS359 and 0.12 μM MK2206) (Figure 3A and Supplementary Figure 7).

### A674563 can overcome FLT3-ligand induced drug resistance in FLT3-ITD/wt co-expressed AML cancer cell lines

We next investigated the ability of A674563 to override drug resistance when FLT3-ITD cells were cultured in the presence of FLT3 ligand. With 10 ng/mL FLT3 ligand stimulation, FLT3-ITD-positive cells were not resistant to A674563. (Figure 3B and Table 3) However, the FLT3 inhibitors TCS359 and MLN518 lost significant activity against FLT3-ITD/FLT3 wt-expressing MOLM13 and MOLM14 cells (6-15 fold), but not against FLT3-ITD homozygous MV4-11 cells (Figure 3B and Table 3) [12]. This is consistent with the report that only FLT3-wt kinase is amenable for the FLT3 ligand stimulation [13]. We then looked at signaling in MOLM14 cells with/without FLT3 ligand. (Figure 3C) As reported previously, phosphorylation of STAT5 was not affected by the presence of FLT3 ligand. However, the inhibitory effects of MLN518 on FLT3 phosphorylation and/or c-MYC expression were decreased upon FLT3 ligand stimulation. Interestingly, there was no significant effect of A674563 treatment in the presence of FLT3 ligand stimulation on the expression of cMYC. (Figure 3C and Supplementary Figure 8) For Akt-mediated signaling, there was an apparent increase of pS6K phosphorylation upon MLN518 treatment in the presence of FLT3 ligand. However, A674563 led to a more potent inhibition of pFOXO1, pS6K and p4EBP1 upon FLT3 ligand stimulation (Figure 3D and Supplementary Figure 9).

**Table 3:** A674563, MLN518 and TCS359 anti-proliferative effect against FLT3 ligand stimulated MOLM13, MOLM14 and MV4-11 cell lines

GI_50_(μM)	A674563	MLN518	TCS359
MOLM-13	0.06	0.042	0.42
MOLM-13+FL(1 ng/mL)	0.061	0.18	> 10
MOLM-13+FL(5 ng/mL)	0.058	0.26	> 10
MOLM-13+FL(10 ng/mL)	0.059	0.27	> 10
MOLM-14	0.18	0.054	10–3
MOLM-14+FL(1 ng/mL)	0.21	0.19	> 10
MOLM-14+FL(5 ng/mL)	0.19	0.2	> 10
MOLM-14+FL(10 ng/mL)	0.18	0.22	> 10
MV4-11	0.075	0.046	0.49
MV4-11+FL(10 ng/mL)	0.078	0.056	1.4

### A674563 inhibits FLT3-ITD-positive primary patient cell proliferation and suppresses tumor progression in a FLT3-ITD/wt heterozygous cell-mediated animal model

**Figure 4:**
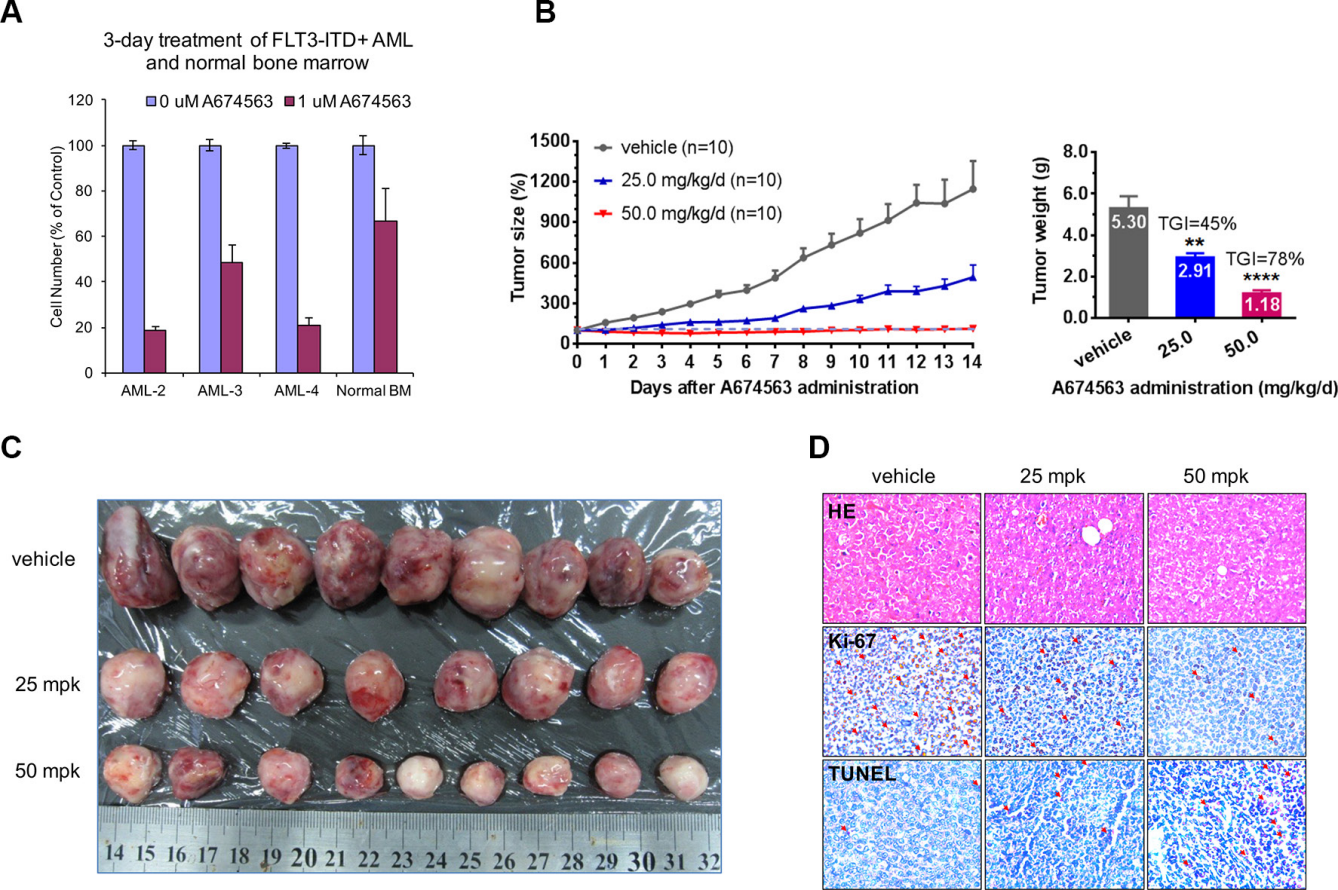
Effect of A674563 on FLT3-ITD-positive primary AML patient cells and *in vivo* anti-tumor activity (**A**) Anti-proliferative effect of A674563 on FLT3-ITD-positive AML patient primary cells and normal bone marrow cells. (**B**) Effect of A674563 on MOLM14 xenograft model. (**C**) Tumor size demonstration by visual measurement. (**D**) Immunohistochemistry staining (HE, Ki-67 and TUNNEL) of tumor tissues.

In FLT3-ITD-positive AML patient primary cells, A674563 exhibited potent anti-proliferation activity at 1 μM and showed apparent selectivity when compared to normal bone marrow cells, which suggests a therapeutic window. (Figure 4A and Supplementary Table 2) In a MOLM14- (FLT3-ITD/FLT3 wt heterozygous) inoculated%. xenograft animal model, A674563 displayed dose-dependent anti-tumor activities. 25 mg/mg/day dose slowed down the tumor progression and resulted in tumor growth inhibition (TGI) of 45. 50 mg/kg/day dosage almost completely suppressed the tumor progression and exhibited an overall tumor growth inhibition (TGI) rate of 78%. (Figure 4B, 4C) KI-67 stain demonstrated that there is a dose-dependent growth inhibitory affect in the tumors (brown color spots number decreasing) and TUNNEL staining confirmed dose-dependent induction of apoptosis in the tumor (brown color spots increasing) (Figure 4D).

## DISCUSSION

Drug resistance is a serious limiting factor for targeted therapy approaches in the clinic [14]. Combination therapy is one of the most effective approaches to overriding this resistance [15]. However, drug-drug interactions and IP issues limit the clinical effectiveness of inclusion of additional drugs in the treatment regimen Rationally controlled multiple-target-single-agent therapy theoretically has advantages to minimize these problems [16].

A674563 has been validated as a selective AKT kinase inhibitor that suppresses tumor growth in the prostate cancer animal models [7]. Previously comprehensive kinome wide selectivity profiling also demonstrates that A674563 has strong binding affinity to FLT3-ITD kinase (Kd: 83 nM compared to 540 nM against FLT3 wt) [17]. It also displays strong binding Kd to other kinases such as AAK1, CIT, CLKs, DYRK1, and PRKs kinases, however currently there is no evidence to support that those kinases are involved in AML. In addition, A674563 exhibited strong binding to ROCK1 kinase as well, which has been implicated to play roles in the c-KIT, FLT3 and BCR-ABL oncogenes mediated myeloproliferative diseases [18]. Whether or not these targets contribute directly or indirectly to the observed anti-FLT3-ITD AML growth activity and FLT3 ligand induced drug resistance would require further mechanistic study. That said, we could not definitely exclude the possibility that target(s) other than AKT/FLT3 contribute to the potent activity of A674563 against FLT3-ITD AML. In addition, although A674563 potently inhibits FLT3-ITD activity in the biochemical assays, FLT3-ITD auto-phosphorylation in the isogenic BaF3 cells as well the downstream target Stat5's phosphorylation in the established AML cell lines MV4-11, it does not potently inhibit FLT3-ITD's auto-phosphorylation in the MV4-11 cells until 5 μM, which indicates that there might be some hidden mechanisms regarding to the FLT3-ITD's auto-phosphorylation and requires further detailed elucidation.

In summary, we have discovered that A674563, a previously reported AKT kinase inhibitor, also demonstrates selective FLT3-ITD kinase activity over FLT3 wt in the biochemical assays, which makes it selectively potent toward FLT3-ITD positive AML cancer cell lines. This dual inhibition efficacy can be recapitulated by the combination of the AKT and FLT3 kinase inhibitors. In addition, A674563 can overcome FLT3 ligand-induced drug resistance. In FLT3-ITD positive AML patient primary cells and a FLT3-ITD-positive xenograft model, A674563 displayed great anti-tumor activity. As clinical resistance can arise in AML patients coharboring FLT3-ITD and FLT3 wt due to the stimulation effects of FLT3 ligand and subsequent hyper-activation of AKT signaling, A674563 as a dual AKT and FLT3-ITD inhibitor could potentially serve as a novel approach to effectively treat FLT3-ITD-positive AML. In addition, A674563 was highly sensitive against FLT3-D835Y mutant, which is another common critical gain of function of mutation of FLT3 in the kinase domain, implying another potential application of A674563.

## MATERIALS AND METHODS

### Inhibitors

A674563, MK2206, GSK690693, TCS359, GDC-0068, AZD5363, AT7867, CCT128930 were purchased from Haoyuan Chemexpress Inc. China.

### Cell lines and cell cultures

The human AML cell lines MV4-11, U937, OCI-AML-3 were purchased from the American Type Culture Collection (ATCC) (Manassas, VA, USA). The FLT3-ITD-expressing lines, MOLM-13, MOLM-14, were provided by Dr. Scott Armstrong, Dana Farber Cancer Institute (DFCI), Boston, MA. The wt FLT3-expressing AML line, NB4 (KRAS A18D), was obtained from Dr. Gary Gilliland. MOLM-13, MOLM-14, U937 and FLT3 mutant isogenic BaF3 cells lines were cultured in RPMI 1640 media (Corning, USA) with 10% fetal bovine serum (FBS) and supplemented with 2% L-glutamine 1% penicillin/streptomycin. MV4-11 was cultured in IDMEM media (Corning, USA) with 10% FBS and supplemented with 2% L-glutamine and 1% penicillin/streptomycin. OCI-AML-3 was cultured in α-MEM media (Corning, USA) with 10% FBS and supplemented with 2% L-glutamine and 1% penicillin/streptomycin. All cell lines were maintained in culture media at 37°C with 5% CO_2_.

### Antibodies and immunoblotting

The following antibodies were purchased from Cell Signaling Technology (Danvers, MA): FLT3 (8F2) Rabbit mAb (#3462), Phospho-FLT3 (Tyr589/591) (30D4) Rabbit mAb (#3464), NF-κB p65 (D14E12) XP^®^ Rabbit mAb (#8242), Phospho-NF-κB p65 (Ser536) (93H1) Rabbit mAb (#3033), Stat5 Antibody (#9363), Phospho-Stat5 (Tyr694) (C71E5) Rabbit mAb (#9314), c-Myc (D84C12) XP^®^ Rabbit mAb (#5605), Akt (pan) (C67E7) Rabbit mAb (#4691), Phospho-Akt (Thr308) (244F9) Rabbit mAb (#4056), Phospho-Akt (Ser473) (D9E) XP^®^ Rabbit mAb (#4060), p44/42 MAPK (Erk1/2) (137F5) Rabbit mAb (#4695), Phospho-p44/42 MAPK (Erk1/2) (Thr202/Tyr204) (D13.14.4E) XP^®^ Rabbit mAb (#4370), GAPDH (D16H11) XP^®^ Rabbit mAb, PARP (46D11) rabbit mAb (#9532), Caspase-3 (8G10) rabbit mAb (#9665), GSK-3β (27C10) Rabbit mAb (#9315), Phospho-GSK-3β (Ser9) (5B3) Rabbit mAb (#9323), FoxO1 (C29H4) Rabbit mAb (#2880), Phospho-FoxO1 (Thr24)/FoxO3a (Thr32)/FoxO4 (Thr28) (4G6) Rabbit mAb (#2599), PRAS40 (D23C7) XP^®^ Rabbit mAb (#2691), Phospho-PRAS40 (Thr246) (C77D7) Rabbit mAb (#2997), 4E-BP1 (#9644), 4E-BP1 (53H11) Rabbit mAb (#9644), p70 S6 Kinase (49D7) Rabbit mAb (#2708), Phospho-p70 S6 Kinase (Thr389) (108D2) Rabbit mAb (#9234). Antibodies were used at 1:1000. Protein lysate preparation and immunoblotting were carried out as previously described.

### FLT3, FLT3-ITD, FLT3-D835Y protein purification

FLT3 wt/FLT3-ITD/FLT3-D835Y cytoplasmic fragment with his tag was cloned into baculovirus expression vector pFASTHT-A. The recombinant bacmid was transfected into SF9 by Cellfectin (Invitrogen). High titer viral stocks were obtained by two rounds of amplification of the virus. The protein was expressed by infecting SF9 cells with high titer viral stocks for 48 h. Cells were harvested and re-suspended in lysis buffer (50 mM Tris pH 7.5, 150 mM NaCl, and 1 mM PMSF). The cells were lysed by ultra sonication and the cell debris was removed by ultracentrifugation. The supernatant was incubated with Ni-affinity beads (GE). The beads were then washed by lysis buffer containing 50–250 mM imidazole. The elute was loaded to superdex 75. The protein was concentrated to 1 mg/ml and aliquots were frozen and stored at −80°C. The protein was used for ADP-Glo™ kinase assay.

### ADP-Glo biochemical assay

The ADP-Glo™ kinase assay (Promega, Madison, WI) was used to screen A674563 for its FLT3 inhibition effects. The kinase reaction system contains 4.95 μL FLT3-WT (40 ng/μL) or FLT3-ITD (40 ng/μL), 0.55 μL of serially diluted A674563, and 5.5 μL FLT3 substrate Poly (4:1 Glu, Tyr) peptide (0.2 μg/μL) (Promega, Madison, WI) with 100 μM ATP (final concentration: 50 μM) (Promega, Madison, WI). The reaction in each tube was started immediately by adding ATP and kept going for an hour under 37°C. After the tube cooled for 5 minutes at room temperature, 5 μL solvent reactions were carried out in a 384-well plate. Then 5 μL of ADP-Glo™ reagent was added into each well to stop the reaction and consume the remaining ADP within 40 minutes. At the end, 10 μL of kinase detection reagent was added into the well and incubated for 30 minutes to produce a luminescence signal. Luminescence signal was measured with an automated plate reader (Perkin-Elmer Envision) and each measurement was performed in triplicate.

### Cell proliferation assay

Cells were grown in 96-well culture plates (3000/well). Various concentrations of drugs were added into plates, DMSO concentrations were kept constant and did not exceed 0.1% of the total volume. Cell proliferation was determined after 72 hours drug treatment. For FLT3 ligand induced drug resistance assay, A674563 and FLT3 inhibitor TCS359, MLN518 were added into the cell culture in the presence of 10 ng/mL FLT3 ligand. Cell viability was measured with the CellTiter–Glo assay (Promega, USA), according to the manufacturer's instructions, and luminescence was measured in a multi-label reader (Envision, PerkinElmer, USA). Data were normalized to control groups (DMSO) and represented by the mean of three independent measurements with standard error < 20%. GI_50_ values were calculated using Prism 5.0 (GraphPad Software, San Diego, CA).

### Kinase kinetic assay

Kinetic analyses of FLT3-ITD were performed using a luminometric kinase assay varying the concentration of ATP using the ADP-Glo reagents (Promega). The serially diluted A674563 and FLT3-ITD (40 ng/μL) were assayed in a reaction (10 μL) containing 40 mM Tris (pH 7.6), 20 mM MgCl_2_, 2 mM MnCl_2_, 50 μM DTT. After 60 min incubation at RT, various concentrations of ATP, 0.2 μg/μL FLT3 substrate Poly (4:1 Glu, Tyr) peptide added and incubated for 60 min at 37°C. The overall rate of reaction is determined as the slope of the decreasing phase of the reaction. Each data point was collected in duplicate and kinetic parameters were obtained using Prism 5.0 (GraphPad Software, San Diego, CA).

### IP kinase assay

The reaction system contains 200 ng FLT3 proteins. The serially diluted A674563 was added to the proteins. After 30 min incubation at RT, ATP (final concentration: 10 μM) was added and incubated for 30 min at 37°C. The reactions were stopped by add 5× sample buffer. Then the Phosphorylation of FLT3 was detected by western-blot using pFLT3 Tyr 589/591 and phosphotyrosine antibody.

### BaF3 isogenic cell line generation

Retroviral constructs for BaF3-FLT3 mutants were made based on the pMSCVpuro (Clontech) backbone. For TEL-FLT3 vector, the first 1 kb of human TEL gene with an artificial myristoylation sequence (MGCGCSSHPEDD) was cloned into pMSCVpuro retroviral vector, followed by a 3xFLAG tag sequence and a stop codon. Then the kinase domain coding sequence of FLT3 was inserted in-frame between TEL and 3× FLAG sequences. For full-length expression vectors, the coding sequences of FLT3 variants were directly cloned in pMSCVpuro vector with a 3X FLAG tag at the C-terminal end. All mutagenesis studies were performed using the QuikChange Site-Directed Mutagenesis Kit (Stratagene) following the manufacturer's instructions. Retrovirus was packaged in HEK293T cells by transfecting FLT3-containing MSCV vectors together with two helper plasmids, virus supernatants were harvested 48 hours after transfection and filtered before infection. Then BaF3 cells were infected with harvested virus supernatants using spinoculation protocol and stable cell lines were obtained by puromycin selection for 48 hours. The IL-3 concentrations in the culture medium were gradually withdrawn until cells were able to grow in the absence of IL-3.

### Combination study

The FLT3-ITD-expressing lines MOLM-13, MOLM14 and MV4-11 were grown in 96-well culture plates (3000/well), respectively. Combination treatment with the FLT3 kinase inhibitor, TCS359, and with the Akt inhibitor, MK2206, was performed. Cell proliferation was determined after treatment with compounds for 72 hours. Cell viability was measured using the CellTiter–Glo assay (Promega, USA), data were normalized to control groups (DMSO) and represented by the mean of three independent measurements with standard error < 20%. CI index was calculated by Calcusyn software (Biosoft, version 2.1), using the Chou-Talalay method [19].

### Cell cycle analysis

MOLM14, MV4-11 cells were treated with serially diluted A674563, TCS359 (1 μM), MK2206 (1 μM) for 24 hours. The cells were fixed in 70% cold ethanol and incubated at −20°C overnight, then stained with PI/RNase staining buffer (BD Pharmingen). Flow cytometry was performed using a FACS Calibur (BD), and results were analyzed by ModFit software.

### Signaling pathway effect examination

MOLM14, MV4-11 cells were treated with DMSO, serially diluted A674563, TCS359 (1 μM), MK2206 (1 μM) for 4 hours. Cells were then washed in PBS and lysed in cell lysis buffer. FLT3, Phospho-FLT3 (Tyr589/591), AKT, Phospho-AKT Ser473, Phospho-AKT Thr308, GSK-3β, Phospho-GSK-3β (Ser9), Phospho-FoxO1 (Thr24), FoxO1, PRAS40, Phospho-PRAS40 (Thr246), STAT5, Phospho-STAT5 (Tyr694), NF-ΚB-P65, Phospho-NF-ΚB-P65 (Ser536), P70S6K, Phospho-P70S6K Thr389, 4EBP1, Phospho-4EBP1 (Thr37/46), ERK, Phospho-p44/42MAPK (Erk1/2) (Thr202/Tyr204), C-Myc and GAPDH antibodies (Cell Signaling Technology) were used for immunoblotting.

For FL addition experiment, MV4-11 cells were treated with FLT3 inhibitors in the presence of 10 ng/mL of FL (FLT3 Ligand, R&D Systems) or absence of FL for 2 hrs. Cells were then washed in PBS and lysed in cell lysis buffer. FLT3, Phospho-FLT3 (Tyr589/591), AKT, GSK-3β, Phospho-GSK-3β (Ser9), Phospho-FoxO1 (Thr24), FoxO1, STAT5, Phospho-STAT5 (Tyr694), P70S6K, Phospho-P70S6K (Thr389), 4EBP1, Phospho-4EBP1 (Thr37/46), C-Myc and GAPDH antibodies (Cell Signaling Technology) were used for immunoblotting.

### Apoptosis effect examination

MOLM14, MV4-11 cells were treated with serially diluted A674563, TCS359 (1 μM), MK2206 (1 μM) for 24 hours. Cells were then washed in PBS and lysed in cell lysis buffer. PARP, Caspase-3, GAPDH antibodies (Cell signaling Technology) were used for immunoblotting.

### Molecular modeling

Based on FLT3 PDB structure (PDB id: 1RJB), which has the DFG-out motif conformation, the DFG-in motif conformation was built by an optimization with DFG-in motif restraining and a short MD released process using Tinker software. Then the released DFG-in motif conformation used as the molecular modeling of FLT3 to dock A674563 compound.

### Colony formation assay

In brief, 1 mL of 3% agarose combined with 1 mL MOLM-13, MOLM-14, MV4-11, PF-382, U937, HL-60 growth media was used as the bottom agar in a 6-well plate. 1000 cells in 1.8 mL growth media was combined with 0.2 μL of 3% agarose solution and 2 μL serially diluted A674563, then plated on top of the bottom layer. Cells were maintained in a humidified 5% CO_2_ incubator at 37°C for 15 days. On the 15th day, the numbers of colonies in each well were counted and each measurement was performed in triplicate.

### Human AML primary cells

Mononuclear cells were isolated from samples from AML patients identified as harboring mutant FLT3. Cells were tested in liquid culture (DMEM, supplemented with 20% FBS) in the presence of different concentrations of inhibitors. All blood and bone marrow samples from AML patients were obtained under approval of the Dana Farber Cancer Institute Institutional Review Board. The ethics committees approved the consent procedure. All studies were performed with ACUC approved protocols at DFCI.

### MOLM-14 xenograft tumor model

Five weeks old female Balb/c-nu mice were purchased from the Shanghai Experimental Center, Chinese Science Academy (Shanghai, China). All animals were housed in a specific pathogen-free facility and used according to the animal care regulations of Hefei Institutes of Physical Science Chinese Academy of Sciences. Prior to implantation, cells were harvested during exponential growth. 10 million MOLM-14 cells in 1640 were formulated as a 1:1 mixture with Matrigel (BD Biosciences) and injected into the subcutaneous space on the right flank of Balb/c-nu mice. Daily oral administration was initiated when MOLM-14 tumors had reached a size of 200 to 400 mm^3^. Animals were then randomized into treatment groups of 10 mice each for efficacy studies. A674563 was delivered daily in a HKI solution (0.5% Methocellulose/0.4% Tween80 in ddH_2_O) by orally gavage. A range of doses of A674563 or their vehicles were administered, as indicated in figure legends. Tumor growth was measured daily after A674563 treatment. Tumor volumes were calculated as follows: tumor volume (mm^3^) = [(W^2^ × L)/2] in which width (W) is defined as the smaller of the two measurements and length (L) is defined as the larger of the two measurements.

### Histological examination

Tumor tissues were fixed in 10% neutral-buffered formalin and embedded in paraffin. Six-micron tissue section were prepared, deparaffinized, dehydrated, and then stained with hematoxylin and eosin (H&E) using routine methods. Commercially available primary antibody to human Ki-67 (ZSGB-BIO, Beijing, China) was used for Ki-67 staining. After heat-induced antigen retrieval, formalin-fixed and paraffin-embedded tumor tissue sections were stained with primary antibody overnight at 4°C. The slides were subsequently incubated with ImmPRES anti-rabbit Ig or anti-mouse Ig (Vector Laboratories, Burlingame, CA) at room temperature for 30 min, stained with peroxidase substrate 3, 3ʹ-diaminobenzidine chromogen (Vector Laboratories), and finally counterstained with hematoxylin. TUNEL staining was assessed using *In Situ* Cell Death Detection Kit (POD) (Roche, Mannheim, Germany) according to the manufacturer's instructions.

## SUPPLEMENTARY MATERIALS FIGURES AND TABLES



## References

[1] Lowenberg B, Downing JR, Burnett A (1999). Acute myeloid leukemia. N Engl J Med.

[2] Markovic A, MacKenzie KL, Lock RB (2005). FLT-3: a new focus in the understanding of acute leukemia. Int J Biochem Cell Biol.

[3] Smith CC, Wang Q, Chin CS, Salerno S, Damon LE, Levis MJ, Perl AE, Travers KJ, Wang S, Hunt JP, Zarrinkar PP, Schadt EE, Kasarskis A (2012). Validation of ITD mutations in FLT3 as a therapeutic target in human acute myeloid leukaemia. Nature.

[4] Wander SA, Levis MJ, Fathi AT (2014). The evolving role of FLT3 inhibitors in acute myeloid leukemia: quizartinib and beyond. Ther Adv Hematol.

[5] Sato T, Yang X, Knapper S, White P, Smith BD, Galkin S, Small D, Burnett A, Levis M (2011). FLT3 ligand impedes the efficacy of FLT3 inhibitors *in vitro* and *in vivo*. Blood.

[6] Chen FL, Ishikawa Y, Kiyoi H, Naoe T (2014). Mechanism of FLT3 Ligand Dependent Resistance to FLT3 Inhibitors. Blood.

[7] Luo Y, Shoemaker AR, Liu X, Woods KW, Thomas SA, de Jong R, Han EK, Li T, Stoll VS, Powlas JA, Oleksijew A, Mitten MJ, Shi Y (2005). Potent and selective inhibitors of Akt kinases slow the progress of tumors *in vivo*. Mol Cancer Ther.

[8] Zarrinkar PP, Gunawardane RN, Cramer MD, Gardner MF, Brigham D, Belli B, Karaman MW, Pratz KW, Pallares G, Chao Q, Sprankle KG, Patel HK, Levis M (2009). AC220 is a uniquely potent and selective inhibitor of FLT3 for the treatment of acute myeloid leukemia (AML). Blood.

[9] Patch RJ, Baumann CA, Liu J, Gibbs AC, Ott H, Lattanze J, Player MR (2006). Identification of 2-acylaminothiophene-3-carboxamides as potent inhibitors of FLT3. Bioorg Med Chem Lett.

[10] Hirai H, Sootome H, Nakatsuru Y, Miyama K, Taguchi S, Tsujioka K, Ueno Y, Hatch H, Majumder PK, Pan BS, Kotani H (2010). MK-2206, an allosteric Akt inhibitor, enhances antitumor efficacy by standard chemotherapeutic agents or molecular targeted drugs *in vitro* and *in vivo*. Mol Cancer Ther.

[11] Okuzumi T, Fiedler D, Zhang C, Gray DC, Aizenstein B, Hoffman R, Shokat KM (2009). Inhibitor hijacking of Akt activation. Nat Chem Biol.

[12] Kelly LM, Yu JC, Boulton CL, Apatira M, Li J, Sullivan CM, Williams I, Amaral SM, Curley DP, Duclos N, Neuberg D, Scarborough RM, Pandey A (2002). CT53518, a novel selective FLT3 antagonist for the treatment of acute myelogenous leukemia (AML). Cancer Cell.

[13] Lee HK, Kim HW, Lee IY, Lee J, Jung DS, Lee SY, Park SH, Hwang H, Choi JS, Kim JH, Kim SW, Kim JK, Cools J (2014). G-749, a novel FLT3 kinase inhibitor, can overcome drug resistance for the treatment of acute myeloid leukemia. Blood.

[14] Ellis LM, Hicklin DJ (2009). Resistance to Targeted Therapies: Refining Anticancer Therapy in the Era of Molecular Oncology. Clin Cancer Res.

[15] Woodcock J, Griffin JP, Behrman RE (2011). Development of novel combination therapies. N Engl J Med.

[16] Jia J, Zhu F, Ma X, Cao Z, Li Y, Chen YZ (2009). Mechanisms of drug combinations: interaction and network perspectives. Nat Rev Drug Discov.

[17] Davis MI, Hunt JP, Herrgard S, Ciceri P, Wodicka LM, Pallares G, Hocker M, Treiber DK, Zarrinkar PP (2011). Comprehensive analysis of kinase inhibitor selectivity. Nat Biotechnol.

[18] Mali RS, Ramdas B, Ma P, Shi J, Munugalavadla V, Sims E, Wei L, Vemula S, Nabinger SC, Goodwin CB, Chan RJ, Traina F, Visconte V (2011). Rho kinase regulates the survival and transformation of cells bearing oncogenic forms of KIT, FLT3, and BCR-ABL. Cancer Cell.

[19] Chou TC (2010). Drug combination studies and their synergy quantification using the Chou-Talalay method. Cancer Res.

