# Supplementary material for: Dual inhibition of AKT/FLT3-ITD by A674563 overcomes FLT3 ligand-induced drug resistance in FLT3-ITD positive AML
Source: Oncotarget. 2016 Apr 11;7(20):29131–42. doi: 10.18632/oncotarget.8675 (PMC5045383; doi:10.18632/oncotarget.8675)
Supplement: Supplementary file 1 [file oncotarget-07-29131-s001.pdf]

# Dual inhibition of AKT/FLT3-ITD by A674563 overcomes FLT3 ligand-induced drug resistance in FLT3-ITD positive AML

## Supplementary Materials

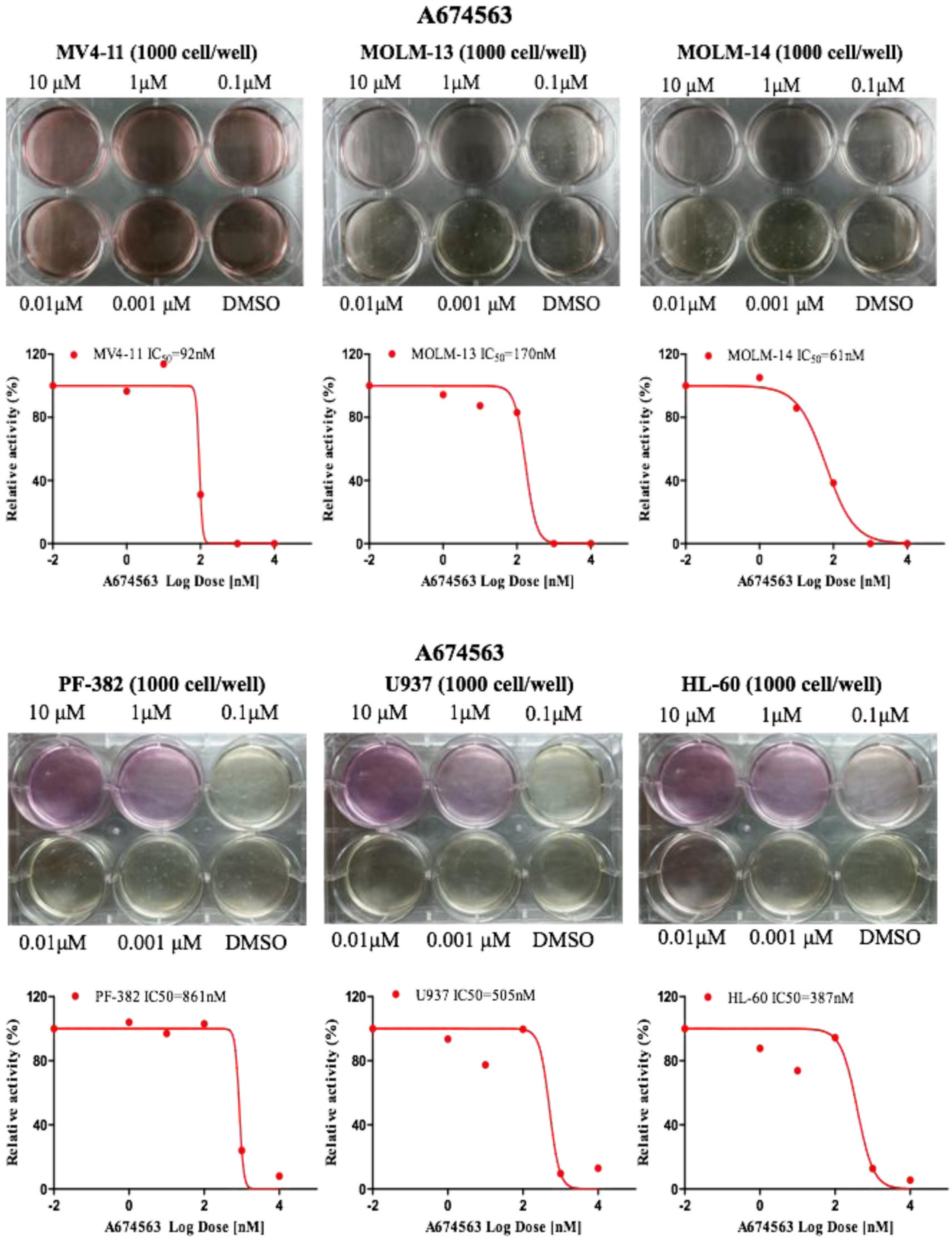


**Supplementary Figure S1: A674563's effect on FLT3-ITD positive AML cells colonogenic formation.**

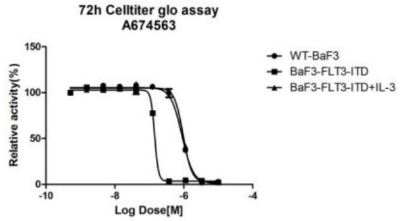


| IC50(μM) | WT-BaF3 | BaF3-FLT3-ITD | BaF3-FLT3-ITD+IL-3 |
|---|---|---|---|
| A674563 | 0.88 | 0.14 | 0.95 |

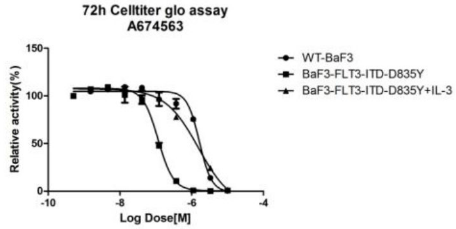


| IC50(μM) | WT-BaF3 | BaF3-FLT3-ITD-D835Y | BaF3-FLT3-ITD-D835Y+IL-3 |
|---|---|---|---|
| A674563 | 1.6 | 0.12 | 1.6 |

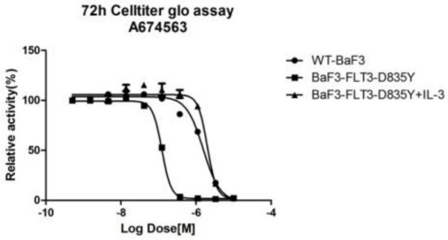


| IC50(μM) | WT-BaF3 | BaF3-FLT3-D835Y | BaF3-FLT3-D835Y+IL-3 |
|---|---|---|---|
| A674563 | 1.6 | 0.12 | 2.0 |

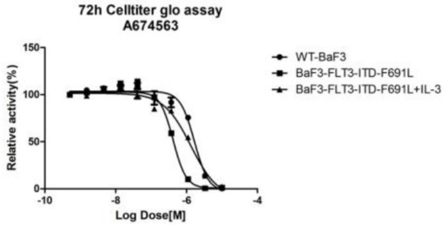


| IC50(μM) | WT-BaF3 | BaF3-FLT3-ITD-F691L | BaF3-FLT3-ITD-F691L+IL-3 |
|---|---|---|---|
| A674563 | 1.7 | 0.42 | 1.3 |

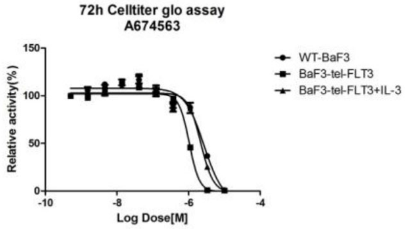


| IC50(μM) | WT-BaF3 | BaF3-tel-FLT3 | BaF3-tel-FLT3+IL-3 |
|---|---|---|---|
| A674563 | 2.7 | 1.0 | 2.2 |

**Supplementary Figure S2: IL-3 rescue experiment on FLT3 wt/mutant transformed BaF3 isogenic cell lines.**

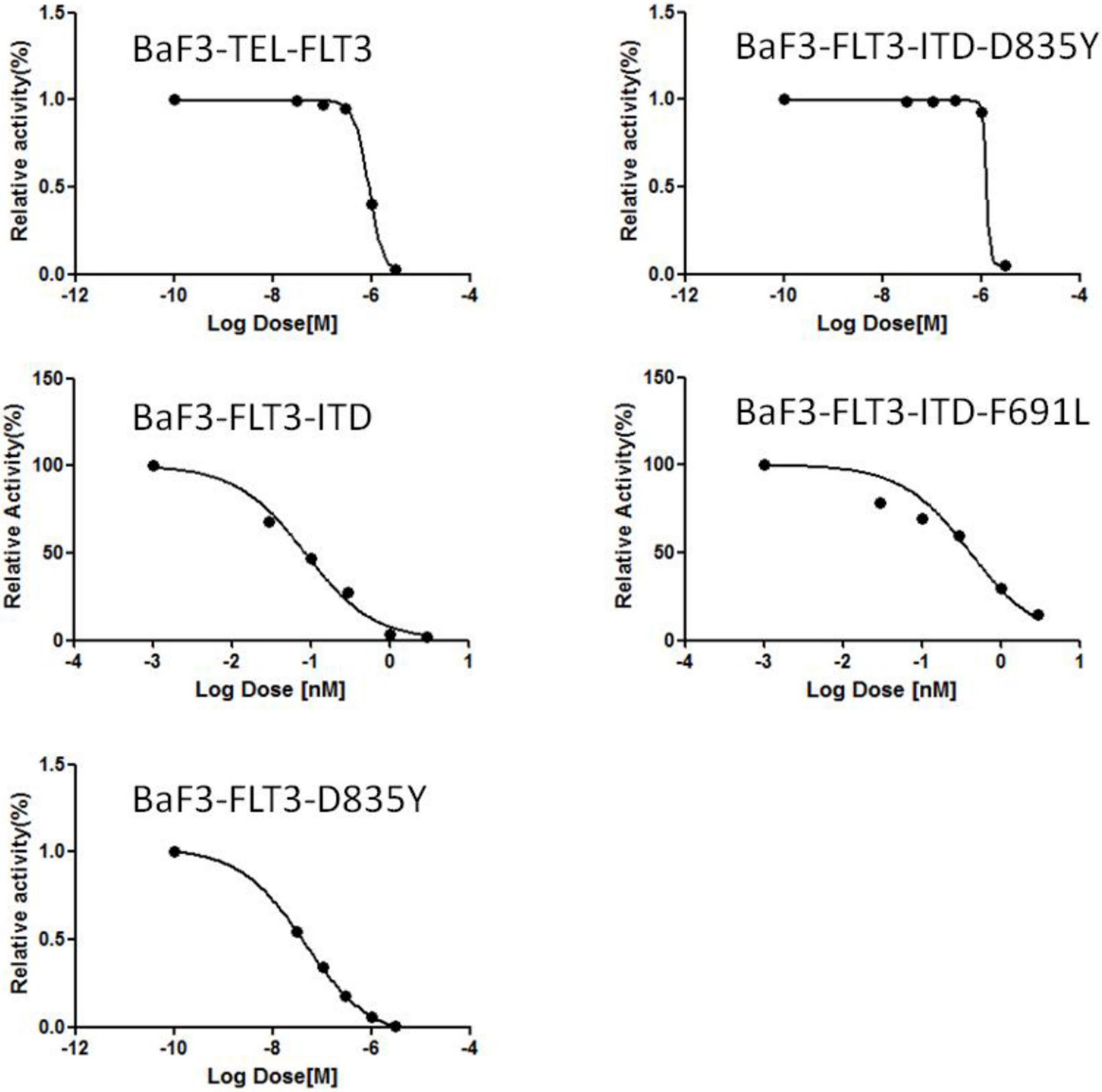


**Supplementary Figure S3: Quantification of A674563's effects on FLT3 phosphorylation.**

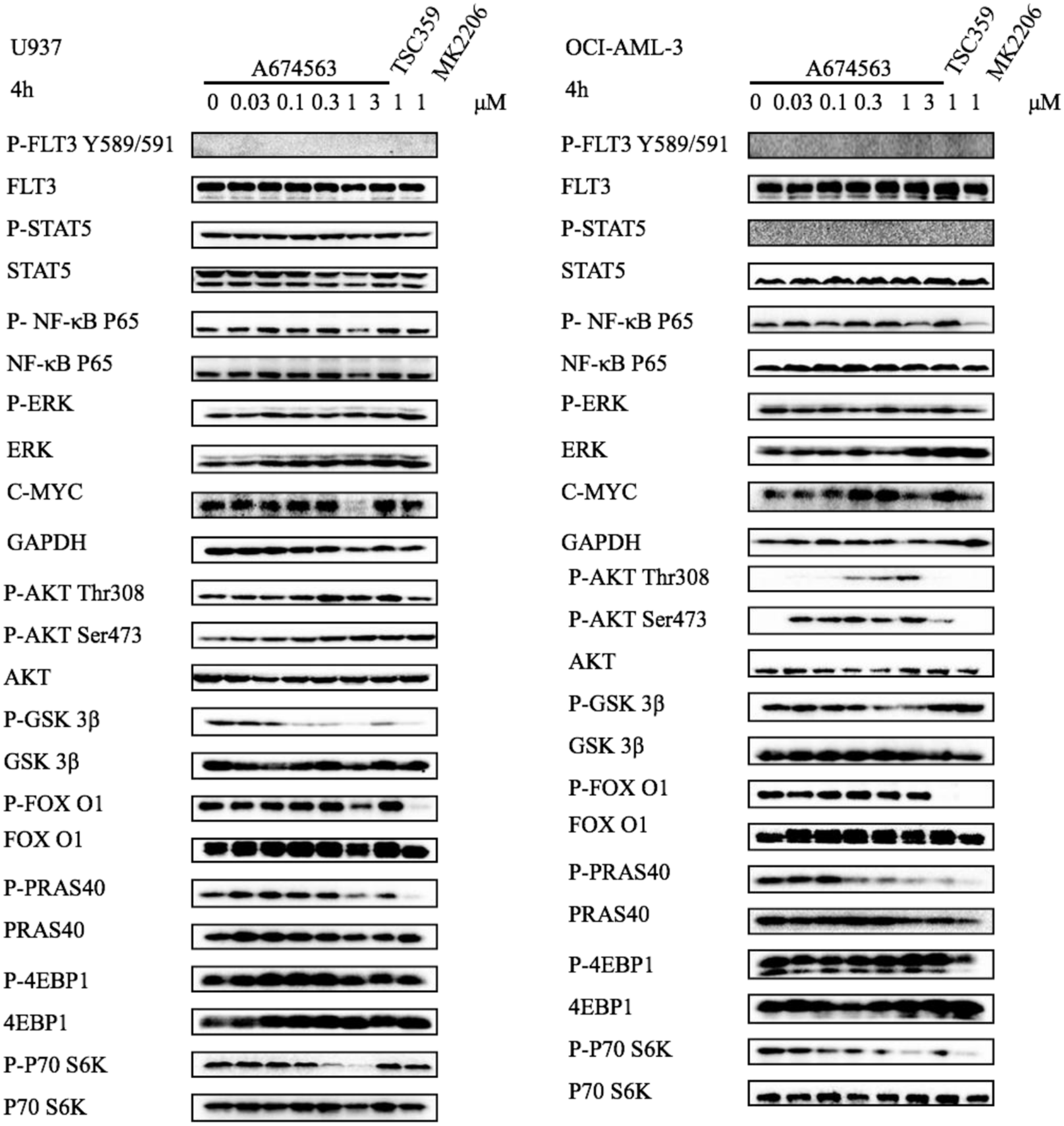


**Supplementary Figure S4: A6754563 effect on FLT3 and Akt mediated signaling pathways in the U937 and OCI-AML-3 cells.**

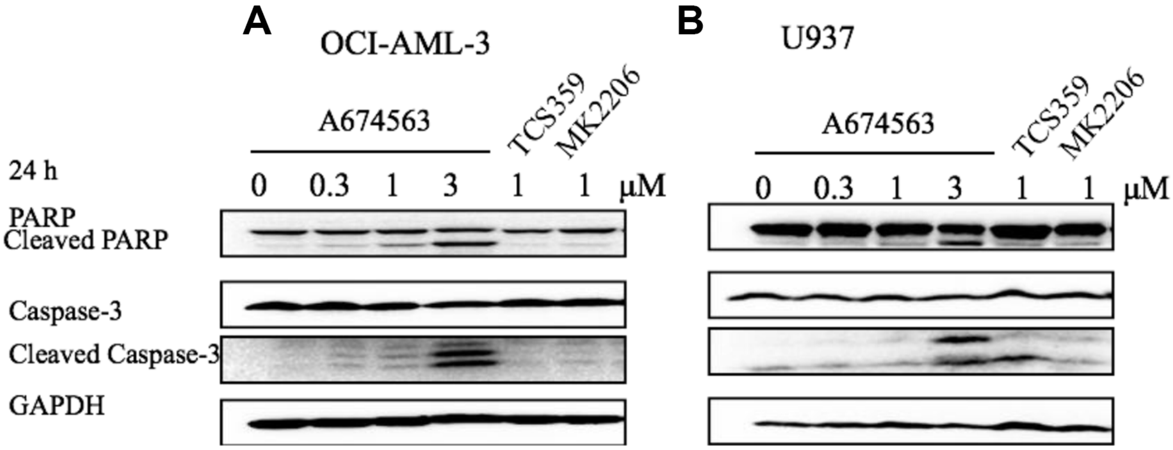


**Supplementary Figure S5: A674563's effect on apoptosis induction in the FLT3 wt cell lines OCI-AML-3 and U937.**

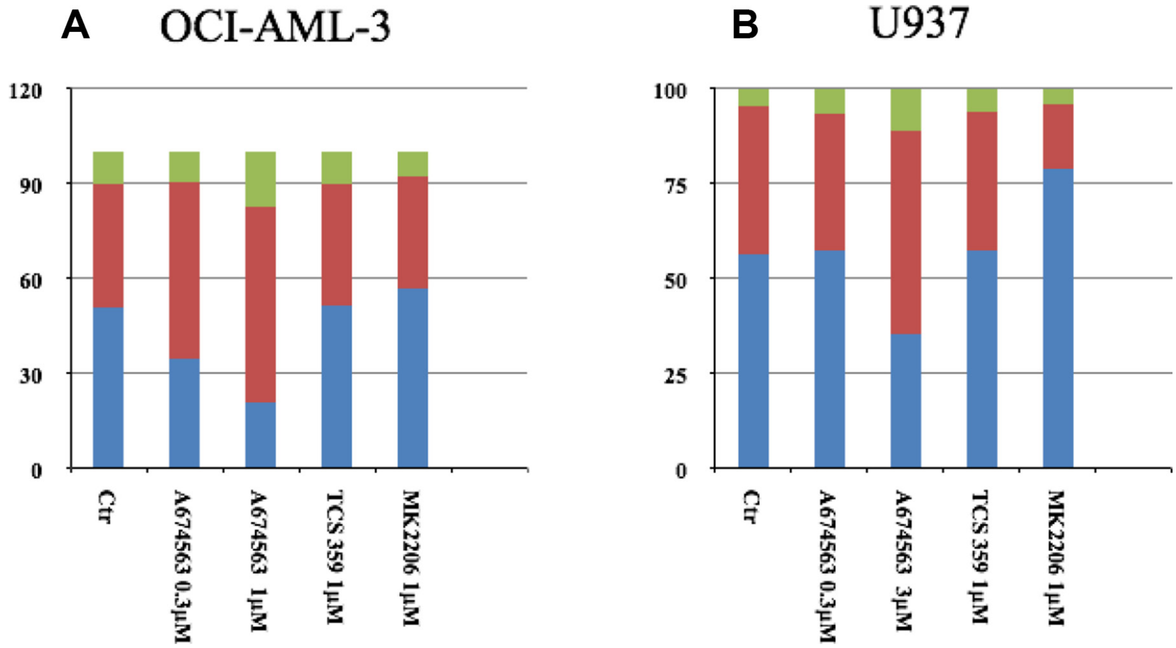


**Supplementary Figure S6: A674563's effect cell cycle progression in the FLT3 wt cell lines OCI-AML-3 and U937.**

MOLM-14

| TCS359 (μM) \ MK2206 (μM) | 0.37 | 0.12 | 0.04 | 0.013 | 0.0045 | 0.000 |
|---|---|---|---|---|---|---|
| 3.30 | 14.4 | 22.8 | 38.7 | 46.9 | 56.0 | 68.5 |
| 1.10 | 19.5 | 36.2 | 46.1 | 60.8 | 66.3 | 71.3 |
| 0.37 | 28.1 | 56.3 | 66.5 | 74.7 | 95.4 | 90.7 |
| 0.12 | 39.8 | 60.4 | 72.4 | 64.5 | 98.2 | 108.5 |
| 0.04 | 37.7 | 58.1 | 73.3 | 84.3 | 90.4 | 99.4 |
| 0.00 | 39.5 | 65.5 | 76.5 | 92.2 | 97.6 | 100.0 |

MOLM-13

| TCS359 (μM) \ MK2206 (μM) | 0.37 | 0.12 | 0.04 | 0.013 | 0.0045 | 0 |
|---|---|---|---|---|---|---|
| 3.3 | 5.5 | 9.3 | 15.4 | 18.1 | 22.1 | 24.0 |
| 1.1 | 10.2 | 15.4 | 31.8 | 34.7 | 34.4 | 32.4 |
| 0.37 | 33.5 | 31.0 | 60.5 | 75.8 | 59.6 | 69.7 |
| 0.12 | 41.3 | 49.3 | 71.0 | 96.2 | 90.7 | 97.6 |
| 0.04 | 39.1 | 47.3 | 75.2 | 95.4 | 87.6 | 104.2 |
| 0 | 38.1 | 59.3 | 76.3 | 94.9 | 89.5 | 100.0 |

MV4-11

| TCS359 (μM) \ MK2206 (μM) | 0.37 | 0.12 | 0.04 | 0.014 | 0.0045 | 0 |
|---|---|---|---|---|---|---|
| 3.3 | 43.2 | 43.5 | 50.9 | 66.4 | 69.3 | 30.4 |
| 1.1 | 52.1 | 53.5 | 59.7 | 58.1 | 64.8 | 43.9 |
| 0.37 | 47.8 | 50.5 | 49.7 | 69.7 | 78.2 | 62.2 |
| 0.12 | 45.2 | 55.5 | 85.5 | 81.0 | 96.3 | 99.0 |
| 0.04 | 51.1 | 55.8 | 63.7 | 84.9 | 85.3 | 108.2 |
| 0 | 79.7 | 88.6 | 98.3 | 105.5 | 112.9 | 100.0 |

**Supplementary Figure S7: Combinational study of TCS359 and MK2206 in FLT3-ITD positive cell lines.**

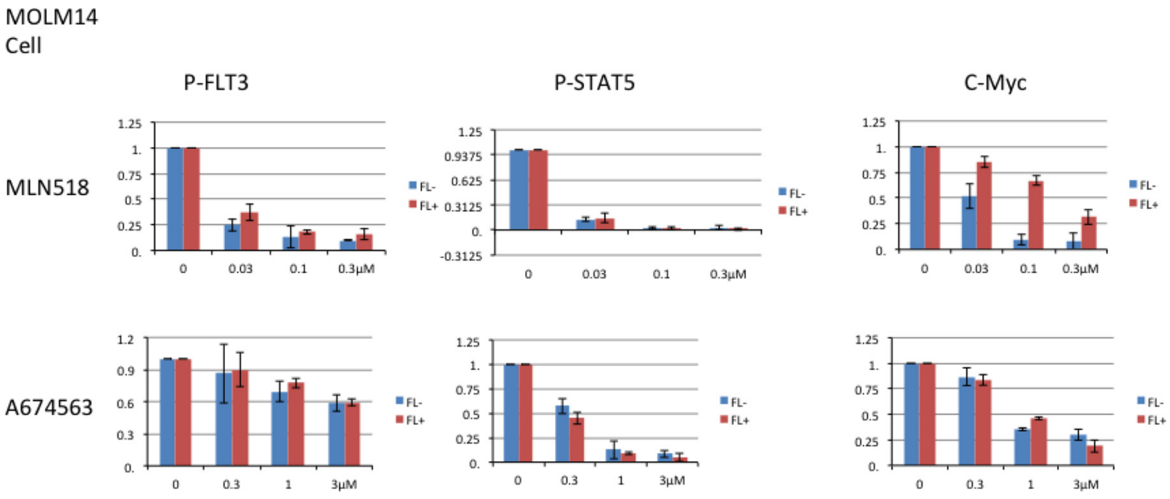


**Supplementary Figure S8: Quantification of FLT3 ligand induced drug resistance in FLT3 mediated signaling**

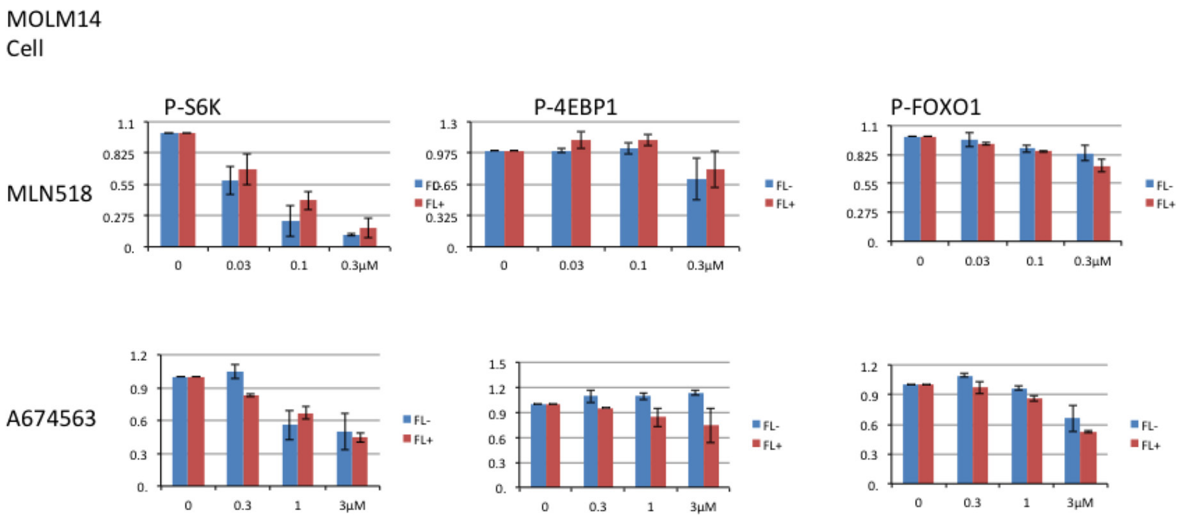


**Supplementary Figure S9: Quantification of FLT3 ligand induced drug resistance in Akt mediated signaling.**

## Supplementary Table S1: Combination effect of FLT3 inhibitor TCS359 and AKT inhibitor MK2206

| MOLM-14 | | | | | |
|---|---|---|---|---|---|
| **Drug** | **DMSO** | **TCS359 (3.3 μM)** | **MK2206 (0.12 μM)** | **Combination** | **CI-index** |
| **Relative activity(%)** | 100 | 68.4 | 65.5 | 22.7 | 0.49 |
| **SD** | 0 | 1.83 | 2.56 | 0.78 | |

| MOLM-13 | | | | | |
|---|---|---|---|---|---|
| **Drug** | **DMSO** | **TCS359 (0.12 μM)** | **MK2206 (0.36 μM)** | **Combination** | **CI-index** |
| **Relative activity(%)** | 100 | 69.7 | 59.3 | 31.0 | 0.41 |
| **SD** | 0 | 4.80 | 4.06 | 1.80 | |

| MV4-11 | | | | | |
|---|---|---|---|---|---|
| **Drug** | **DMSO** | **TCS359 (0.12 μM)** | **MK2206 (0.12 μM)** | **Combination** | **CI-index** |
| **Relative activity(%)** | 100 | 98.9 | 88.6 | 55.5 | 0.29 |
| **SD** | 0 | 0.64 | 4.79 | 3.39 | |

## Supplementary Table S2: AML patient primary cell line information

| Sample ID | Gender | Age | Genetic information |
|---|---|---|---|
| AML2 | M | 69 | 90% bone marrow blasts; 23 K WBC count; crit: 24; 5% peripheral blasts; previous therapy: azacytidine, cytarabline, high dose Ara-c; cytogenetics: normal; mutations: SRSF2 (54%), ASXL1 (46%), RUNX1 (39.4%), TET2 (ins) (46%), TET2 (point mutation) (2.8%), TET2 (del) (3.5%), FLT3-ITD (51 aa). |
| AML3 | F | 66 | 75% bone marrow blasts; 5 K WBC count; crit: 24; 13% peripheral blasts; previous therapy: azacytidine; cytogenetics: trisomy 21, ring chromosome 18; mutations:NPM1 (41%), DMT3 mutation (45%), IDH1 (2.5%), PHF6 (32%), FLT3-ITD (66 aa, 18 aa). |
| AML4 | F | 68 | 5–10% bone marrow blasts; 3.9 K WBC count; crit: 34; 0% peripheral blasts; previous therapy: 3 + 7 chemotherapy, sorafenib, high dose Ara-c, mitoxantrone/etoposide/cytarabine, allogeneic stem cell transplant; cytogenetics: normal; mutations: STAG2 (14.2%), TET2 (5.5%). |

## Supplementary Table S3: cell cycle analysis data

| MOLM14 | %G0–G1 | %S | %G2–M | MV4-11 | %G0–G1 | %S | %G2–M |
|---|---|---|---|---|---|---|---|
| Ctr | 44.19 | 44.06 | 11.74 | Ctr | 56.61 | 31.93 | 11.46 |
| A674563 0.3 μM | 47.08 | 41.65 | 11.27 | A674563 0.3 μM | 68.69 | 28.7 | 2.61 |
| A674563 3 μM | 53.87 | 23.49 | 22.65 | A674563 3 μM | 68.62 | 22.82 | 8.56 |
| TSC359 1 μM | 55.04 | 35.88 | 9.08 | TSC359 1 μM | 73.62 | 23.81 | 2.57 |
| MK2206 1 μM | 63.9 | 28.81 | 7.3 | MK2206 1 μM | 64.92 | 27.39 | 7.69 |
| GSK690693 1 μM | 54.52 | 35.7 | 9.78 | GSK690693 1 μM | 60.48 | 32.52 | 7 |
